# Optimizing the Morphology and Solidification Behavior of Fe-Rich Phases in Eutectic Al-Si-Based Alloys with Different Fe Contents by Adding Mn Elements

**DOI:** 10.3390/ma17164104

**Published:** 2024-08-19

**Authors:** Lei Luo, Yingchun Tang, Xiao Liang, Yanqing Su, Youwei Zhang, Huasheng Xie

**Affiliations:** 1 School of Materials Science and Technology, Nanjing University of Aeronautics and Astronautics, Nanjing 210016, China; liangxiao@nuaa.edu.cn; 2 Institute Materials Physics, Helmholtz-Zentrum Hereon, Max-Planck-Str. 1, D-21502 Geesthacht, Germany; 3 National Key Laboratory for Precision Hot Processing of Metals, School of Materials Science & Engineering, Harbin Institute of Technology, Harbin 150001, China; suyq@hit.edu.cn; 4 China Academy of Machinery Shenyang Research Institute of Foundry Co., Ltd., Shenyang 110022, China; zhangyw@chinasrif.com (Y.Z.); xiehs@chinasrif.com (H.X.)

**Keywords:** high Fe content, Al-Si-based alloys, Fe-rich phases, morphology evolution, solidification behavior

## Abstract

A high Fe content easily produces Fe-rich phases with a harmful morphology, resulting in a huge detrimental effect on the properties and recycling ability of Al-Si alloys. Therefore, finding ways to effectively transform Fe-rich phases to form a beneficial phase or shape is of great significance. Accordingly, Al-Si-based alloys with Fe contents ranging from 0.1 wt.% to 2.0 wt.% were modified by different Mn additions. Moreover, experiments combined with simulations were utilized to comprehensively analyze the mechanism of Mn on the morphology and microstructural evolution of Fe-rich phases from different perspectives. The current findings determine that adding different Fe contents changes the phase-transition reactions in alloys. Without Mn, and by increasing the Fe content from 0.1 wt.% to 2.0 wt.%, the Fe-rich phases gradually convert from a skeleton-shaped *α*-Al_8_Fe_2_Si (<0.25 wt.%) to *β*-Al_9_Fe_2_Si_2_ with a fibrous (0.5 wt.%), needle-like (1.0 wt.%) and plate-like shape without curvatures (2.0 wt.%). The maximum length and mean aspect ratio increase from 12.01 μm to 655.66 μm and from 1.96 to 84.05, while the mean curvature decreases from 8.66 × 10^−2^ μm^−1^ to 8.25 × 10^−4^ μm^−1^. The addition of 0.35 wt.% Mn promotes a new Chinese-character and petal-shaped *α*-Al_15_(FeMn)_3_Si_2_, with an atomic ratio of Fe and Mn of 1:1 when the Fe content is lower than 0.5 wt.%, while it transforms to *β*-Al_15_(FeMn)_3_Si_2_ with an atomic ratio of 5:1, presenting as a refined plate-like shape with a certain curvature, as the Fe content increases to 2.0 wt.%. Mn alters the phase reactions and increases the threshold of the Fe content required for *β*-Al_15_(FeMn)_3_Si_2_, limiting the formation and growth of them simultaneously in time and space. The enrichment of Mn atoms and solute diffusion at the growth front of *β*-Al_15_(FeMn)_3_Si_2_, as well as the strong atomic-binding ability, can deflect the growth direction of *β*-Al_15_(FeMn)_3_Si_2_ for it to have a certain curvature. Additionally, the enriched Mn atoms easily form *α*-Al_15_(FeMn)_3_Si_2_ and cause the long *β*-Al_15_(FeMn)_3_Si_2_ to be broken and refined to further reduce the damages caused to the alloy’s performance. Ultimately, the maximum length and mean aspect ratio can be effectively reduced to 46.2% and 42.0%, respectively, while the mean curvature can be noticeably increased by 3.27 times with the addition of Mn.

## 1. Introduction

Eutectic Al-Si-based alloys (whose Si content ranges from 10 wt.% to 13 wt.%) [1], such as the ZL102, 108, and 109 Al alloys, are important and widely used as piston heads and pin bosses in engines, due to their excellent comprehensive performance, including a high strength-to-weight ratio, a low thermal expansion coefficient, good thermal conductivity, and eminent wear and corrosion resistance [2,3,4,5]. Iron is the most common and inevitably harmful impurity that damages the properties and recycling ability of Al-Si-based alloys by forming large quantities of detrimental morphologically Fe-rich phases through each cycle process [6,7]. Concretely, a high Fe content can easily promote *β*-Al_9_Fe_2_Si_2_ to produce a large plate-like shape without any curvatures [8], which will continuously grow in length, resulting in blocked feeding channels and weak melt flows between dendrites, as well as a reduced feeding capacity and increased shrinkage defects [9,10]. These coarsen phases usually act as the locations of high stress concentrations and the origins of micro-cracks, seriously reducing the mechanical properties and recycling ability of alloys [11,12,13]. Accordingly, finding ways to effectively eliminate the harmful effects caused by Fe-rich phases in Al-Si alloys with high Fe contents is of great significance. To this end, many approaches have been proposed from the perspective of solidification behaviors, alloy melt regulations, and microstructural optimizations, respectively, such as increasing the cooling rate to improve the solubility of iron in the aluminum matrix, bringing out a reduction in the risk of iron [14]. However, this method is more suitable for low-dimension materials, such as thin films and powders, rather than the casting Al-Si alloys with large sizes [15]. Another method is to break or separate the Fe-rich phases into alloys by adding physical fields, such as ultrasonic treatments, electromagnetic field treatments, high vacuum die casting, and so on, and to refine their morphology [16,17,18,19,20]. However, these methods are difficult to control and usually require supporting devices, which increases the complexity and cost of casting preparations. Some studies suggest that Fe-rich phases can be changed by subsequent heat treatments, so as to re-melt and recrystallize them to achieve phase-refinement effects [21,22,23,24]. But unfortunately, since the morphology of Fe-rich phases is very large in alloys with a high iron content, ideal effects cannot be achieved by heat treatments, and this method becomes a palliative rather than a cure. Contrastively, adding extra elements (such as Mn, Cr, Co, V, Sr, and Ce) is the simplest and most cost-effective method [25,26,27,28,29,30] that can directly modify Fe-rich phases naturally. For instance, the addition of Mn elements can effectively vary from *β*-Al_9_Fe_2_Si_2_ to *α*-Al_8_Fe_2_Si, and the effects depend on the content ratio of iron to manganese in Al-Si-based alloys [31,32,33,34]. Currently, a large amount of the literature [29,30] has mainly revealed the influences of the Mn and Fe contents on the type and shape of different Fe-rich compounds for improving the mechanical properties of materials. Nevertheless, these research studies focus on alloys with a lower iron content, usually less than 0.5 wt.%. Moreover, the variations in solidification behaviors, phase transformation processes, and Fe-rich phase evolutions of the alloys induced by a higher Fe content (>0.5 wt.%) and different Mn additions have not been systematically studied and reported on. Furthermore, the relevant mechanisms are indistinct and unexplained.

In this regard, in the current work, the effects of different Fe contents and Mn additions on the evolution of Fe-rich intermetallics are systematically studied to optimize the microstructure, property, and circularity of Al-12Si alloys. Noteworthily, a high Fe content (2.0 wt.%) was used to magnify the formation and growth behaviors of Fe-rich intermetallics to further analyze, more intuitively, the modification effects of manganese elements to the transformation of Fe-rich phases during solidification. In addition, we comprehensively explain the mechanisms from the perspective of the phase-transition reactions, solute diffusion, and solidification behaviors of alloys during the solidification process, respectively. Our objective is to explore the optimization effects of Mn on Fe-rich intermetallics in Al-Si-based alloys with a high Fe content and to reveal the relevant influencing mechanisms to thereby provide an innovative solution and a theoretical basis for the high performance and cyclability of Al-Si alloys.

## 2. Methods

### 2.1. Material Preparation

Al-12Si-based alloys were used as model alloys in this study and were prepared by using raw materials of pure aluminum (with a high purity of 99.99 wt.%), Al-30 wt% Si, Al-50 wt.% Cu, Al-10 wt.% Ni, Al-10 wt.% Fe, and Al-20 wt.% Mn master alloys (all compositions quoted in this work are in wt.% unless stated otherwise). The chemical composition of near-eutectic Al-Si alloys with different Fe levels are an Al-12Si-1Cu-0.5Ni-xFe alloy and an Al-12Si-1Cu-0.5Ni-0.35Mn-xFe alloy (x = 0.1, 0.25, 0.5, 1.0, 1.5, and 2.0), respectively. The research in this paper mainly focuses on the ZL108 and ZL109 alloys with a silicon content of about 12 wt.%, so a part of the Cu and Ni elements were added to achieve a product that is closer to these aluminum alloy systems. Meanwhile, in this study, we also wanted to study whether low-temperature precipitated phases are affected differently during the growth of Fe-rich phases, so as to facilitate a more optimal design of the alloy in a subsequent study. Each sample was melted at 1000 K in a resistance furnace and was degassed with high-purity argon. In the casting process, the pure aluminum was first melted. After the aluminum was completely melted, the other elements were added into the alloy melt, separately. When all the alloy material was melted, after holding for 15 min, the alloy melt was poured into a cylindrical mold to carry out an air-cooling process. The mold was 20 mm in diameter and 100 mm in length and was preheated at 723 K. The chemical compositions of the samples are shown in Table 1.

### 2.2. Measurement and Analysis Methods

The specimens were ground and polished with diamond suspensions and were all cut from the same position of the prepared casting alloys in a dimensional shape of 10 × 5 × 5 mm^3^. The samples for microstructure observation were etched in a mixed aqueous HF solution (5%) for 2 s. Subsequently, these specimens were rinsed in alcohol and distilled water, successively, and were then rapidly dried and preserved for later use. A crystal structure analysis of the precipitated phase and component identification were performed through high-resolution transmission electron microscopy (HR-TEM, Tecnai G2 F20, FEI, Columbia, MD, USA). The microstructure characterizations were analyzed by using scanning electron microscopy (SEM, Quanta 200FEG, FEI, Columbia, MD, USA) and optical microscopy (OM, GX53, Olympus, Tokyo, Japan). Additionally, energy-dispersive spectroscopy (EDS, Quanta 200FEG, FEI, Columbia, MD, USA); X-ray diffraction (XRD, Empyrean, Panalytical, Almelo, The Netherlands); and X-ray photo-electron spectroscopy (XPS, ESCALAB 250Xi, ThermoFisher, Waltham, MA, USA) were used together to determine the chemical composition and nature of the precipitation phases in the microstructure. Moreover, a differential scanning calorimeter (DSC; QMS403D-Is 50, NETZSCH, Selb, Germany) was used by heating the samples with a rate of 10 K/min to analyze the melting temperatures of different phases.

### 2.3. Simulations and Calculations

The solidification path, solidification sequence, and phase diagram of the different samples were calculated by the Pandat software 2018 (CompuTherm LLC, Middleton, WI, USA). A statistical analysis of the maximum length, maximum width, mean aspect ratio, and mean curvature of the Fe-rich intermetallics were performed by the Image-Pro v6.0 software (Media Cybernetics, Inc., Rockville, MD, USA). With respect to the mean aspect ratio and mean curvature of the Fe-rich intermetallics, the measurements and calculations were repeated 50 times to obtain an average value of which the standard deviation was approximately 2%. In addition, the measurements of the maximum length and maximum width were counted by taking 50 sets of data to select the maximum values, and the error of the measurement was about 2%.

## 3. Results

### 3.1. Changes of the Solidification Sequence of Al-12Si-1Cu-0.5Ni-xFe Alloys by Adding Mn Elements

**Figure 1:**
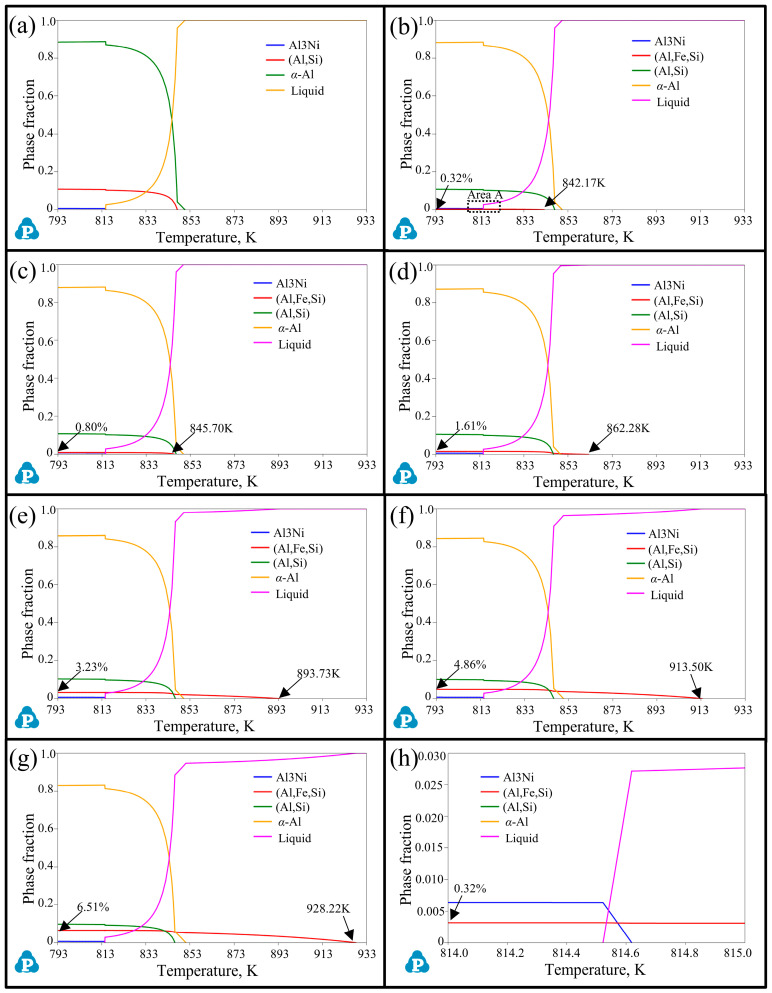
Solidification sequence of Al-12Si-1Cu-0.5Ni-xFe alloys from 793 K (completely solid) to 933 K (completely liquid). (**a**–**g**) are alloys with an Fe content of 0, 0.1, 0.25, 0.5, 1.0, 1.5, and 2.0 wt.%, respectively. (**h**) Enlarged images of Area A in (**b**).

**Figure 2:**
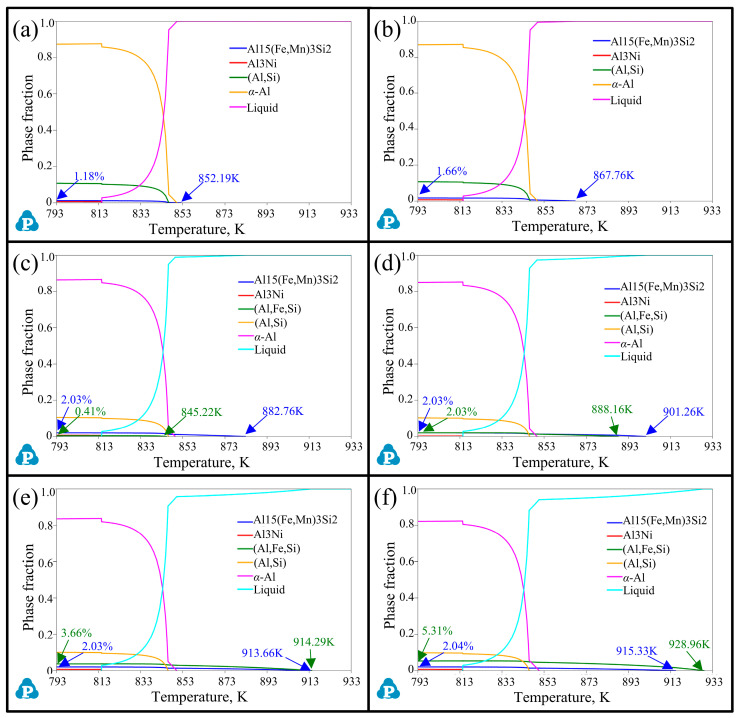
Solidification sequence of Al-12Si-1Cu-0.5Ni-0.35Mn-xFe alloys from 793 K (completely solid) to 933 K (completely liquid). (**a**–**f**) are alloys with an Fe content of 0.1, 0.25, 0.5, 1.0, 1.5, and 2.0 wt.%, respectively.

The addition of Fe and Mn elements can cause noticeable variations in the solidification sequence of alloys, leading to changes in the precipitation temperature and volume fraction of phases. In this regard, the solidification sequence of Al-12Si-1Cu-0.5Ni-xFe alloys and Al-12Si-1Cu-0.5Ni-0.35Mn-xFe alloys (x = 0, 0.1, 0.25, 0.5, 1.0, 1.5, and 2.0) from 793 K (completely solid) to 933 K (completely liquid) were calculated by using the Pandat software, as exhibited in Figure 1 and Figure 2 and Table 2. In alloys without the addition of Mn (Figure 1 and Table 2), when the Fe content was 0 wt.%, the matrix phase *α*-Al first precipitated at 850.61 K as the temperature decreased, followed by the monatomic silicon phase (847.20 K) and the Al_3_Ni phase (814.76 K). The monatomic silicon phase formed a binary Al-Si eutectic phase (Al,Si) with the *α*-Al [35]. As a result, the phase fraction of *α*-Al, the (Al,Si) eutectic phase, and the Al_3_Ni phase were 88.48%, 10.83%, and 0.681%, respectively. When the Fe element was added, a new precipitated (Al,Si,Fe) phase appeared, whose precipitation temperature gradually increased with the promotion of the Fe content, accompanied by a certain decrease in the other phases. When the Fe content increased from 0.1 wt.% to 2.0 wt.%, the precipitation temperature and phase fraction of the (Al,Si,Fe) phases elevated from 842.17 K to 928.22 K and from 0.32% to 6.51%, respectively. The (Al,Si,Fe) phase began to preferentially precipitate at 862.28 K before *α*-Al (849.61 K), and the phase fraction became 1.61% when the Fe content reached 0.5 wt.%. The phase fraction of *α*-Al and the (Al,Si) eutectic phase dropped due to an increasing formation of the (Al,Si,Fe) phase, while the amount of Al_3_Ni phases slightly raised (Table 2).

Additionally, the change of the solidification sequences in alloys containing a 0.35 wt.% Mn element by adding different Fe contents are shown in Figure 2. The addition of the Mn element contributed to a new precipitated (Al,Si,Fe,Mn) phase with a high proportion of manganese atoms forming and replacing the (Al,Si,Fe) phase in contrast to the alloys without any Mn content. Specifically, when the Fe content was very low (0.1 wt.%), under the action of the Mn element, an (Al,Si,Fe,Mn) phase preferentially formed at 852.19 K instead of *α*-Al at 850.15 K. When the Fe content reached 0.5 wt.%, the (Al,Si,Fe) phase containing a lower proportion of manganese atoms appeared again. Meanwhile, the precipitation temperature and phase fraction of both the (Al,Si,Fe,Mn) and (Al,Si,Fe) phases were promoted by continuously adding the iron element. When the Fe content was 1.0 wt.%, the precipitation temperature of the (Al,Si,Fe,Mn) phase (901.26 K) was higher than the (Al,Si,Fe) phase (888.17 K), and the phase fraction of them were close. However, as the Fe content increased to 1.5 wt.%, the precipitation temperature and phase fraction of the (Al,Si,Fe) phase (914.29 K, 3.66%) were higher than those of the (Al,Si,Fe,Mn) phase (913.66 K, 2.037%). In general, when the Fe content was low (<0.5 wt.%), the (Al,Si,Fe) phase was not formed in the alloys due to the addition of Mn and was replaced by the (Al,Si,Fe,Mn) phase. However, when the Fe content exceeded 0.5 wt.%, the (Al,Si,Fe) phase reappeared and surpassed the (Al,Si,Fe,Mn) phase in precipitation temperature and phase fraction. In this condition, although the precipitation temperature of the (Al,Si,Fe,Mn) phase still increased marginally, the volume fraction was hardly promoted (Table 2).

By comparing Figure 1 and Figure 2 and Table 2, it can be concluded that an improvement in the Fe content can observably change the solidification sequence of alloys, leading to the formation of Fe-rich phases and increasing the precipitation temperature and phase fraction of them. Contrastively, the addition of Mn can form the (Al,Si,Fe,Mn) phase to effectively prevent and replace the emergence of the (Al,Si,Fe) phase when the Fe content is low (<0.5 wt.%). Compared with alloys without the Mn element, when the Fe content is higher than 1.0 wt.%, although this inhibition ability of Mn to the appearance of the (Al,Si,Fe) phase begins to decrease, it still has obvious advantages and the potential to reduce the total amount of the (Al,Si,Fe) phase harmful to the properties of the alloy.

### 3.2. Microstructural Evolution of Al-12Si-1Cu-0.5Ni-xFe Alloys by Adding Mn Element

**Figure 3:**
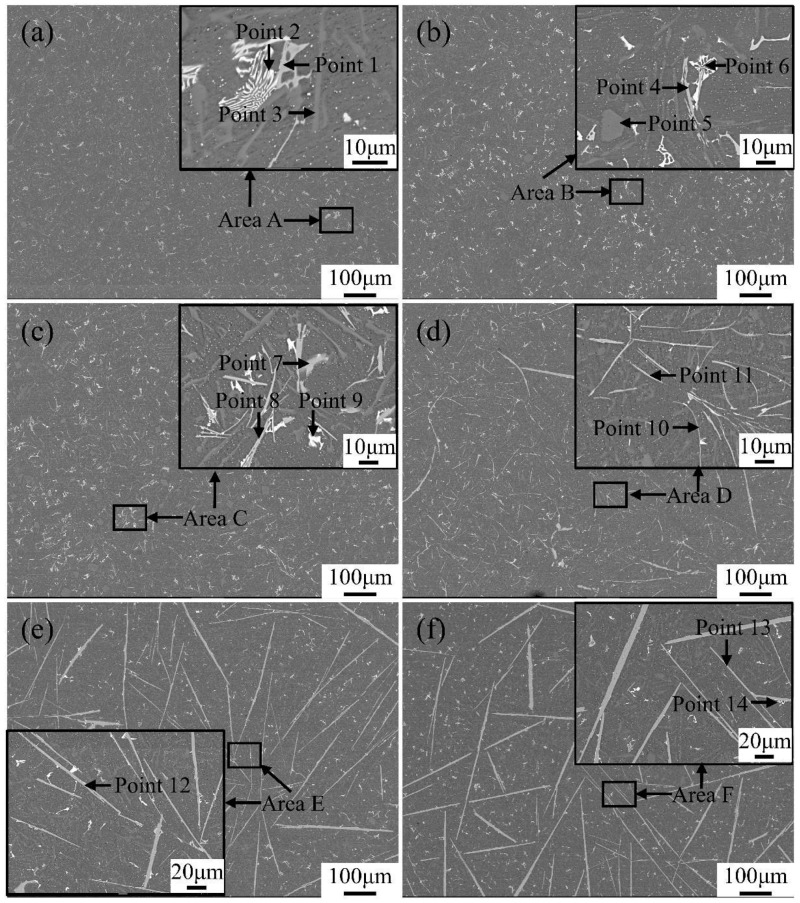
Scanning electron micrographs of Al-12Si-1Cu-0.5Ni-xFe alloys. (**a**–**f**) are the samples numbered #1–#6, respectively.

**Figure 4:**
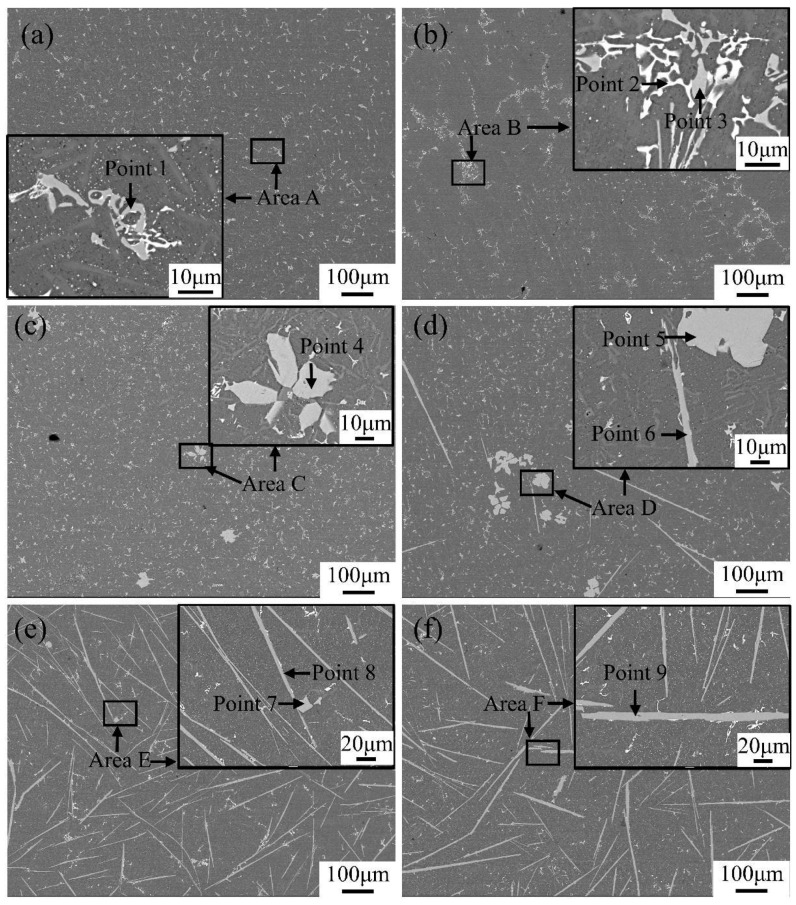
Scanning electron micrographs of Al-12Si-1Cu-0.5Ni-0.35Mn-xFe alloys. (**a**–**f**) are the samples numbered #7–#12, respectively.

The eutectic point of the Al-Si binary alloy is 12.6 wt.% of the silicon content. The Al-12Si-based alloys used in this experiment belong to the near-eutectic range. Therefore, the solidification behavior of alloys is easily changed by the addition of Fe and Mn. In order to facilitate observations, the different samples were analyzed by SEM and EDS. The variations in the microstructure and phase composition of alloys with different additions of the Fe and Mn elements are demonstrated in Figure 3 and Figure 4, Table 3 and Table 4.

The EDS results in Table 3 and Table 4 show that the microstructure of the alloys were dominated by *α*-Al, primary Si particles, Al-Si binary eutectic phases, Fe-rich phases, and (Al,Cu,Ni) phases. A grey-white phase with a skeleton shape, filamentous shape, needle-like shape, and plate-like shape, shown as Points 1, 4, 7, 8, 10, 11, 12, and 13 in Figure 3 and Points 1, 3, 4, 5, 6, 7, 8, and 9 in Figure 4, were the Fe-rich phases. Additionally, the regular block phase was the primary Si particles (Point 5 in Figure 3), whose nucleation was on the Fe-rich phases reported from previous research [35]. The dark-gray phases that were different from the *α*-Al matrix phase were the Al-Si binary eutectic phases (Point 3 in Figure 3), which presented a short acicular morphology and were uniformly dispersed in the matrix [36]. Compared with the aforementioned phases, the other brighter phases with a grid shape were the (Al,Cu,Ni) phases, represented as Points 2, 6, 9, and 14 in Figure 3 and Point 2 in Figure 4, which contained more Cu and Ni elements with a large atomic number and which were distributed uniformly in the alloys and which were often accompanied by Fe-rich intermetallics. Furthermore, it should be noted that the (Al,Cu,Ni) phase was composed of Al_3_Ni and Al_2_Cu, and, for the sake of brevity, the intermetallic mixture will be referred to as the (Al,Cu,Ni) phase, hereinafter.

Focusing on the Fe-rich phases, as demonstrated in Figure 3, it was confirmed that when the Fe content was lower than 0.25 wt.% in the alloys without any Mn elements, the (Al,Si,Fe) phase with a skeleton shape appeared (Points 1 and 4 in Figure 3a,b, respectively). As the Fe content increased to 0.5 wt.%, the volume fraction and size of the (Al,Si,Fe) phase began to grow (Figure 3c). Under this condition, the (Al,Si,Fe) phase with a skeleton shape (Point 7) and with a filamentous shape having a certain curvature (Point 8) occurred in the alloy simultaneously. Meanwhile, in the alloys with a 1.0 wt.% Fe content, the morphology of the (Al,Si,Fe) phase was no longer skeleton-like but was mainly filamentous (Point 10) and needle-like (Point 11) in shape. Moreover, when the Fe content improved to 2.0 wt.%, the (Al,Si,Fe) phase grew along the direction of length and thickness and formed a plate-like shape without any curvatures (Point 13), resulting in a sharp decrease in the aspect ratio for the Fe-rich phases. Moreover, the atomic ratio of the Si and Fe in the (Al,Si,Fe) phases having a skeleton shape was 2 to 1, while this ratio was 1 to 1 in the filamentous-, needle-like-, and plate-like-shaped Fe-rich phases [37].

Contrastively, the (Al,Si,Fe,Mn) phase appeared in the alloys with the addition of Mn, as the results in Figure 4 and Table 4 show. Moreover, when the Fe content was lower than 0.5 wt.%, the small-sized (Al,Si,Fe,Mn) phase with a Chinese character shape formed, represented as Point 1 in Figure 4a and Point 3 in Figure 4b, respectively, and transformed to an aggregated petal shape as the Fe content increased to 0.5 wt.% and 1.0 wt.% (Figure 4c,d). In addition, by raising the Fe content from 1.0 wt.% to 2.0 wt.%, the (Al,Si,Fe,Mn) phase containing a lower proportion of Mn atoms formed again, whose morphology transformed from the Chinese character and petal shape to a needle-like and plate-like shape (Figure 4d–f). In this process, the volume fraction and the maximum length and width of the (Al,Si,Fe,Mn) phase containing a lower content of Mn atoms slowly rose and grew, compared to the alloys without any Mn elements. Noteworthily, the plate-like-shaped (Al,Si,Fe,Mn) phase appeared to be broken and refined and presented a certain curvature. Additionally, the atomic ratio of Fe and Mn in the (Al,Si,Fe,Mn) phases represented as Chinese character-shaped and petal-shaped were close to 1 to 1, while the atomic ratios were close to 5 to 1 in the needle-like- and plate-like-shaped (Al,Si,Fe,Mn) phases (Table 4).

**Figure 5:**
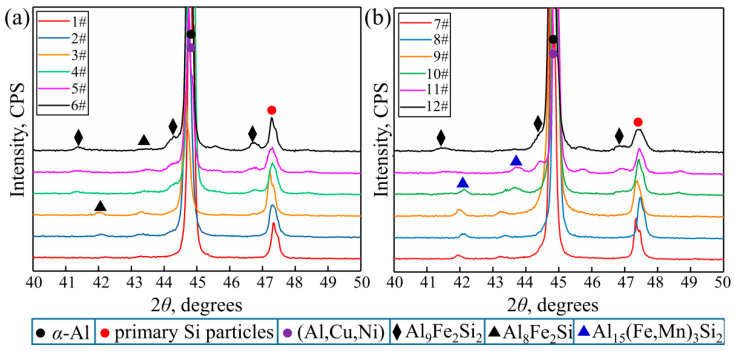
XRD maps of different samples. (**a**) 0 Mn. (**b**) 0.35 wt.% Mn.

To further determine the phase composition of the different alloys, XRD tests performed, and the results are shown in Figure 5. It should be noted that because the Fe-rich phases between 40 and 50 degrees were relatively concentrated in the XRD results, this region was studied emphatically. Additionally, the XRD results in this paper are only used as qualitative means, not as quantitative means. Finally, the analysis was carried out by a combination of XRD, SEM, EDS, TEM and other methods. It was concluded from the results in Table 3 and Table 4 and Figure 5 that the main precipitate phases in the alloys were mainly *α*-Al (matrix phase), primary Si particles, (Al,Cu,Ni) phases, and Fe-rich phases. With regard to the Fe-rich phases, these were mainly presented as *α*-Al_8_Fe_2_Si and *β*-Al_9_Fe_2_Si_2_ in the alloys without the addition of Mn, where Al8FeSi emerged as a skeleton shape and *β*-Al_9_Fe_2_Si_2_ surfaced as a filamentous, needle-like, and plate-like shape. Meanwhile, the addition of the Mn element resulted in a new Fe-rich phase of Al_15_(FeMn)_3_Si_2_ appearing in the alloys. When the atomic ratio of Fe and Mn was close 1 to 1, the shape of Al_15_(FeMn)_3_Si_2_ was a Chinese character shape and petal shape and transformed to a needle-like- and plate-like-shaped Fe-rich phase with an atomic ratio of about 5 to 1 as the Fe content continuously increased. Moreover, the results in Figure 5a also indicate that when the Fe content was low (<0.5 wt.%), the Fe-rich phases were mainly *α*-Al_8_Fe_2_Si phases, which began to transform to *β*-Al_9_Fe_2_Si_2_ as the Fe content reached 0.5 wt.%. Meanwhile, the addition of Mn can effectively promote the precipitation of Al_15_(FeMn)_3_Si_2_ (Figure 5b) and can restrain the formation of *β*-Al_9_Fe_2_Si_2_. As a result, the formation of *β*-Al_9_Fe_2_Si_2_ was replaced by the (Al,Si,Fe,Mn) phase with an atomic ratio of Fe and Mn of 5 to 1. In addition, this type of (Al,Si,Fe,Mn) phase needs to be produced in alloys with a higher Fe content (>1.5 wt.%). In other words, the addition of Mn increased the Fe content threshold required for the formation of the (Al,Si,Fe,Mn) phase with an atomic ratio of Fe and Mn of 5 to 1 in the alloys, and this fact is more conducive to the optimization of the microstructure and the improvement of the performance of alloys [38].

### 3.3. Changes in Morphology of Fe-Rich Phases in Al-12Si-1Cu-0.5Ni-xFe Alloys by Adding Mn Element

**Figure 6:**
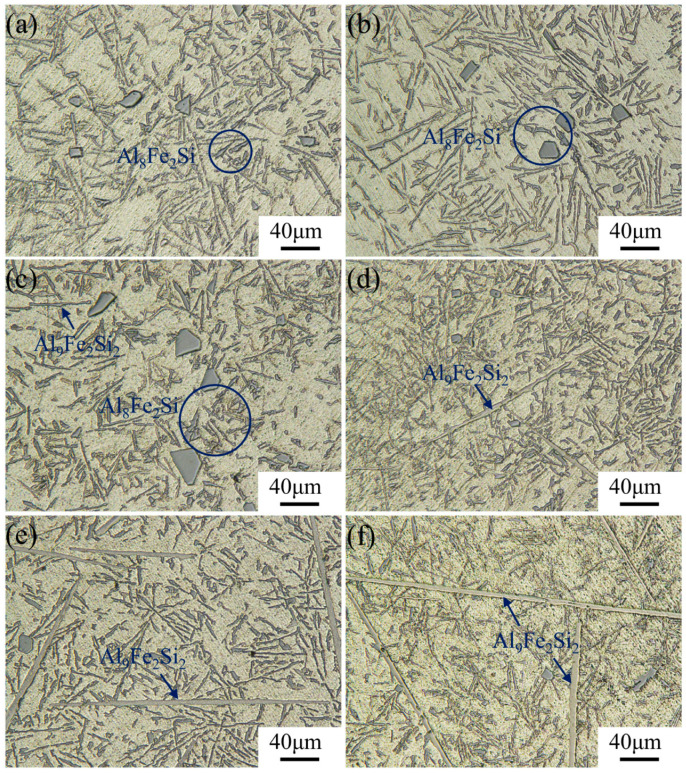
Metallographic pictures of Al-12Si-1Cu-0.5Ni-xFe alloys obtained using an optical microscope. (**a**–**f**) are the samples numbered #1–#6, respectively.

**Figure 7:**
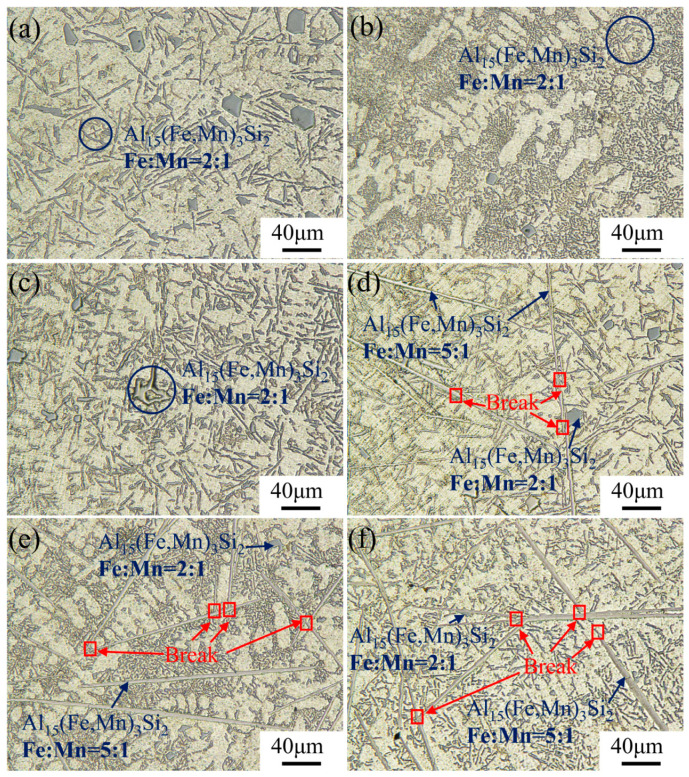
Metallographic pictures of Al-12Si-1Cu-0.5Ni-0.35Mn-xFe alloys obtained using an optical microscope. (**a**–**f**) are the samples numbered #7–#12, respectively.

**Figure 8:**
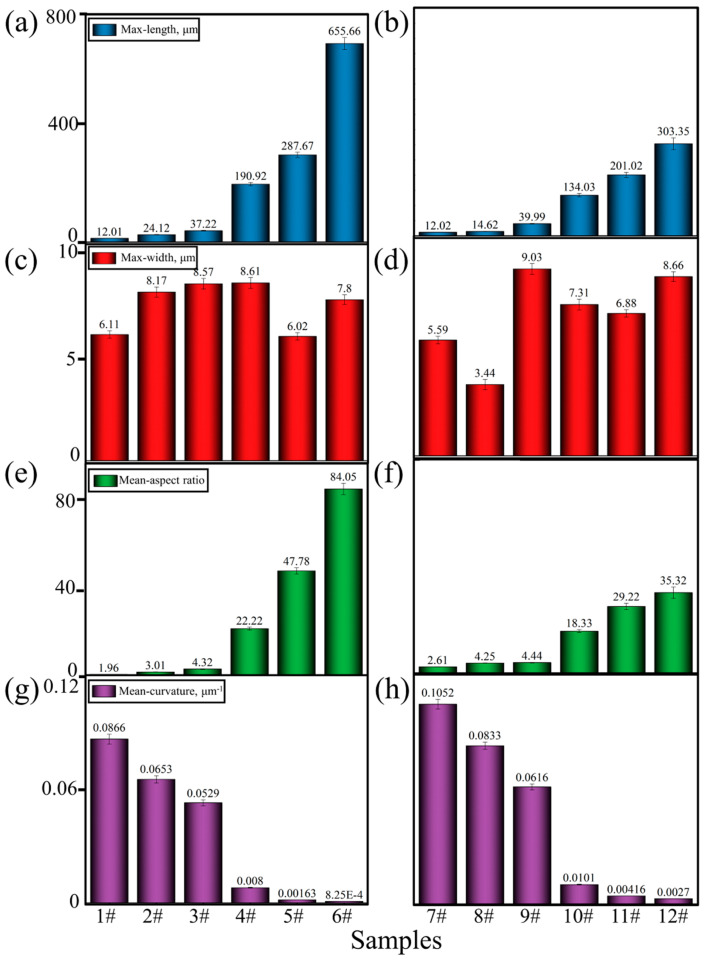
Maximum length, maximum width, mean aspect ratio, and mean curvature of Fe-rich phases in different samples. (**a**,**c**,**e**,**g**) are the samples numbered #1–#6. (**b**,**d**,**f**,**h**) are the samples number #7–#12.

It can be determined from Figure 3 and Figure 4 that the difference in the addition of Fe and Mn elements can lead to significant variations of the Fe-rich phases in alloys. In order to further qualitatively analyze the regulation mechanism of the morphology of Fe-rich phases, OM tests were carried out on each group of patterns, as shown in Figure 6 and Figure 7. In addition, the Image-Pro software was used to count and calculate the length, width, curvature, and other relevant information for the Fe-rich phases, as shown in Figure 8.

Specifically, with the enhancement of the Fe content, the Fe-rich phase in alloys without the addition of Mn gradually changed from skeletal (0.1–0.5 wt.% Fe content) to fibrous in shape with a certain curvature (0.5–1.0 wt.% Fe content). During the continuous increase in Fe content, the Fe-rich phase grew along the direction of length and width and transformed to a needle-like and plate-like shape. In this progress, the growth rate in the length direction was much faster than that in the width direction, resulting in a significant increase in the aspect ratio for the morphology of Fe-rich phases. From the previous analysis, it was clear that the Fe-rich phase with a skeletal shape was *α*-Al_8_Fe_2_Si (Figure 6a–c), and the fibrous-shaped phase was the refined *β*-Al_9_Fe_2_Si_2_ (Figure 6c). Meanwhile, the needle-like and plate-like shape with a higher aspect ratio were the grown *β*-Al_9_Fe_2_Si_2_ phase, which had a huge negative effect on the properties of the alloys (Figure 6d–f). When the Mn element was added, the formation of Al_15_(FeMn)_3_Si_2_ was effectively promoted. Al_15_(FeMn)_3_Si_2_ with a ratio of 1 to 1 for Fe and Mn atoms appeared as Chinese-character shaped and petal shaped (Figure 7). Interestingly, the petal-shaped Al_15_(FeMn)_3_Si_2_ produced a polymerization phenomenon that inhibited the formation and growth of the Al_15_(FeMn)_3_Si_2_ phase with a lower atomic proportion of Mn [39]. As the Fe content increased to more than 1.0 wt.% and 2.0 wt.%, the plate-like-shaped Al_15_(FeMn)_3_Si_2_ phase with a ratio of 5 to 1 for Fe and Mn atoms appeared (Figure 7d–f). In this process, the Al_15_(FeMn)_3_Si_2_ phase having a certain curvature slowly grew and appeared to be broken and refined (Figure 7d–f). The reason for this is that the modification of Mn restricted the growth of the Al_15_(FeMn)_3_Si_2_ phases and broke them and had the potential to make them distribute uniformly in the matrix as a strengthening phase. In general, with the addition of Mn, the morphology of the Fe-rich phase in alloys with a high Fe content can be refined accordingly. Meanwhile, Mn can significantly increase the threshold of the Fe content required for the transformation of Fe-rich phases with a beneficial morphology (a Chinese character shape and petal shape) to a harmful morphology (a needle-like and plate-like shape).

Moreover, the maximum length, maximum width, mean aspect ratio, and mean curvature of the Fe-rich phases in each group were calculated by the Image-Pro software, as shown in Figure 8. It was found that, regardless of whether Mn was added, the increase in the Fe content aggrandized the length, width, and aspect ratio but reduced the curvature of the Fe-rich phases during the transformation from a skeletal, Chinese character, and petal shape to a fibrous, needle-like, and plate-like shape. In contrast to alloys without Mn elements, the addition of 0.35 wt.% Mn effectively promoted the precipitation and growth of Al_15_(FeMn)_3_Si_2_ to restrict and replace the formation of the *β*-Al_9_Fe_2_Si_2_ phase. Furthermore, in the alloys with a higher content of Fe (2.0 wt.%), the Mn element still limited the growth of the Al_15_(FeMn)_3_Si_2_ phase, with a ratio of 5 to 1 for Fe and Mn atoms, along the length direction to some extent, causing it to have a certain curvature and to be more easily broken. Finally, the big-sized Al_15_(FeMn)_3_Si_2_ phases were also refined and evenly distributed in the matrix, which had the potential to improve the performance of the alloy as the strengthening phase of the matrix [40,41]. According to the statistical results shown in Figure 8, it can be seen that in alloys without Mn, the improvement of the Fe content from 0.1 wt.% to 2.0 wt.% violently increased the maximum length and mean aspect ratio of the Fe-rich phases from 12.01 μm to 655.66 μm (Figure 8a) and from 1.96 to 84.05 (Figure 8e), respectively, while sharply decreasing the curvature from 8.66 × 10^−2^ μm^−1^ to 8.25 × 10^−4^ μm^−1^ (Figure 8g). These changes were most obvious in the alloys with an Fe content that exceeded 0.5 wt.%. During this process, the maximum width did not change greatly (Figure 8c,d).

Summarily, by increasing the Fe content from 0.1 wt.% to 2.0 wt.% in the alloys with the addition of 0.35 wt.% Mn, the maximum length and mean aspect ratio of the Fe-rich phases increased from 12.02 μm to 303.35 μm (Figure 8b) and from 2.61 to 35.32 (Figure 8f), respectively. Meanwhile, the mean curvature decreased from 1.05 × 10^−1^ μm^−1^ to 2.70 × 10^−3^ μm^−1^ (Figure 8h). Compared to the alloys without Mn, the addition of Mn effectively modified the Fe-rich phase to obtain a certain curvature even at a high Fe content and made it break and refine during its growth process. Ultimately, under the same Fe-content conditions, the maximum length and the aspect ratio can be effectively reduced to 46.2% and 42.0%, respectively, while the curvature can be noticeably increased by 3.27 times with the addition of Mn.

## 4. Discussion

The effects of Fe and Mn on the evolution of Fe-rich phases in alloys were discussed and analyzed scientifically from different perspectives, including phase-transition reactions and solute diffusion processes.

### 4.1. The Perspective of Phase-Transition Reactions during the Solidification Process in Different Alloys

**Figure 9:**
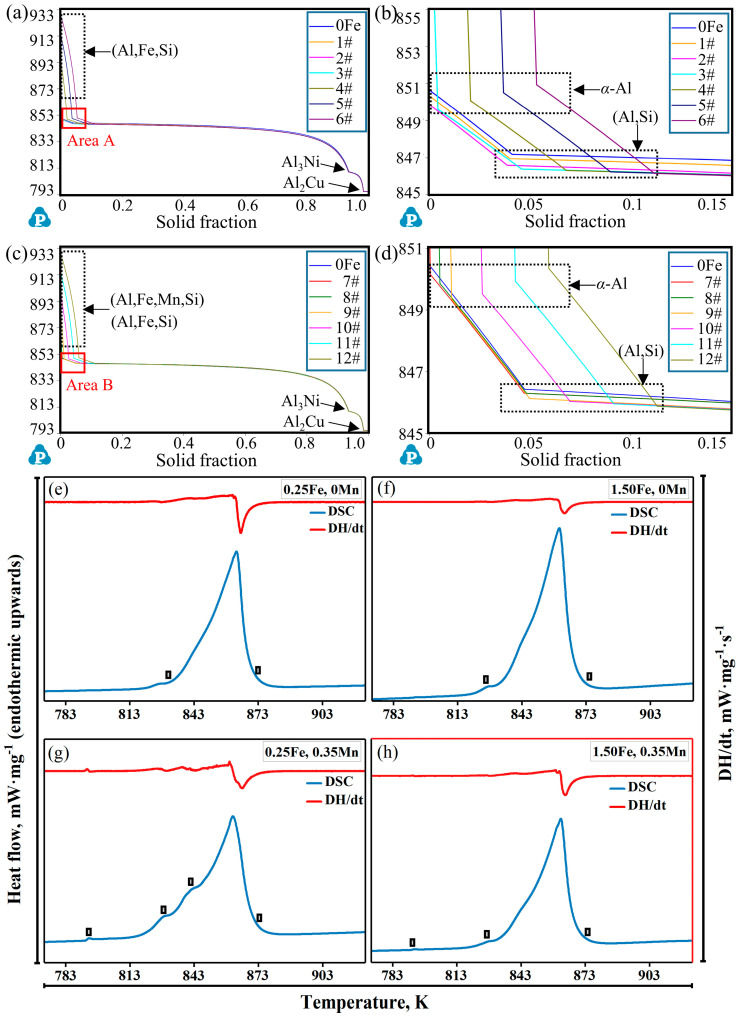
Equilibrium solidification paths and the results of the DSC tests of the different samples. (**a**,**c**) are the solidification paths. (**b**,**d**) are the enlarged images of Area A in (**a**) and Area B in (**c**), respectively. (**e**–**h**) are the results of the DSC tests. Note: The black rectangles in (**e**–**h**) represent the corresponding positions of endothermic peaks.

**Figure 10:**
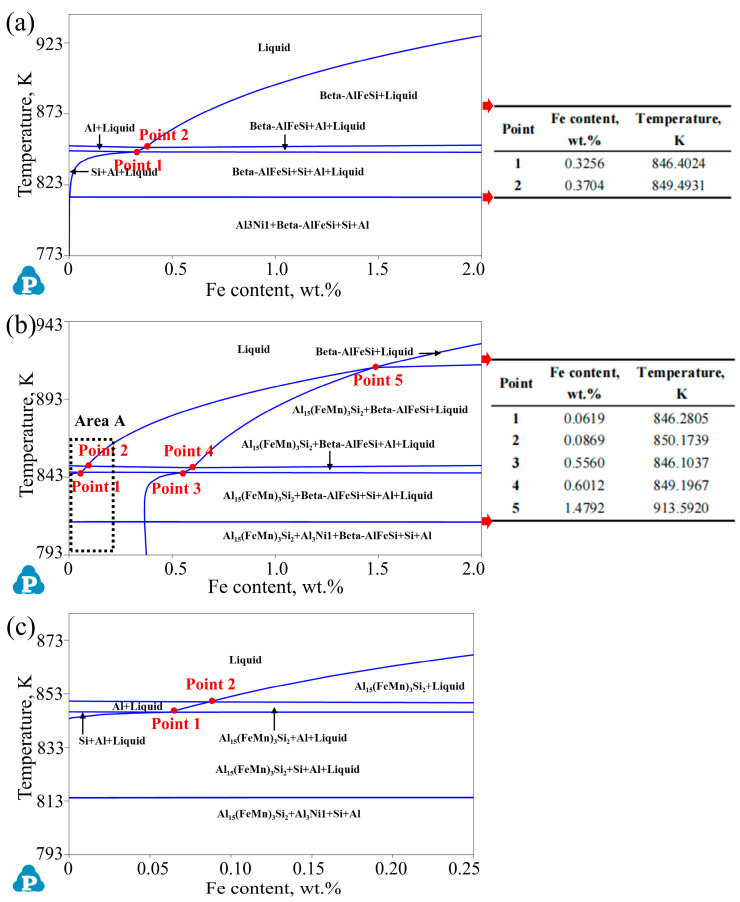
Phase diagrams of different samples. (**a**) Al-12Si-1Cu-0.5Ni-xFe (x = 0~2.0 wt.%) alloys without Mn elements. (**b**) Al-12Si-1Cu-0.5Ni-0.35Mn-xFe (x = 0~2.0 wt.%) alloys. (**c**) Enlarged images of Area A in (**b**).

In order to explore the action mechanism of Fe and Mn on Fe-rich phases, the equilibrium solidification paths and phase diagrams of different alloys were calculated by the Pandat software, as shown in Figure 9a–d and Figure 10. And DSC tests were carried out, as shown in Figure 9e–h, to verify the results.

It was revealed that in the alloys without the addition of Mn, when the Fe content was less than 0.5 wt.%, there were 4 obvious inflection points in the solidification paths corresponding to *α*-Al, (Al,Si), Al3Ni, and Al2Cu, respectively, according to the order of occurrence. At the point where the slope of the curve changes, the Fe-rich phase appears. When the Fe content increased to more than 0.5 wt.%, there were 5 inflection points in the solidification process. And the new inflection points were represented as the (Al,Fe,Si) phases, which preferentially appeared compared to the other phases as the temperature of the alloys decreased (Figure 9a). The rise in the Fe content substantially promoted the precipitation temperature of the (Al,Fe,Si) phase, causing the precipitation temperature of *α*-Al to first decrease from 850.61 K (0 wt.%) to 849.61 K (0.5 wt.%) and then increase to 851.12 K (2.0 wt.%). Meanwhile, the precipitation temperature of the (Al,Si) phase decreased from 847.20 K to 846.13 K (Figure 9b), and the precipitation temperature of Al_3_Ni and Al_2_Cu exhibited little changes. Based on a previous study, it can be determined that the (Al,Cu,Ni) was composed of Al_3_Ni and Al_2_Cu phases. When Mn was added to the alloys, the (Al,Fe,Si) phases were replaced by the (Al,Fe,Mn,Si) phases. In contrast, when the Fe content was 0.1 wt.%, the (Al,Fe,Mn,Si) phase began to form, of which the precipitation temperature was higher than *α*-Al (Figure 9c,d). There were 5 inflection points in the solidification curves of the alloys, corresponding to the (Al,Fe,Mn,Si) phase, *α*-Al, (Al,Si), Al_3_Ni, and Al_2_Cu, respectively, according to the precipitation sequence. Similarly, when the Fe content improved from 0 to 2.0 wt.%, the precipitation temperature of (Al,Fe,Mn,Si) increased from 852.19 K to 928.96 K, while the *α*-Al first decreased from 850.61 K (0 wt.%) to 849.38 K (0.5 wt.%) and then increased to 850.59 K (2.0 wt.%). Similarly, the precipitation temperature of the (Al,Si) phase decreased, and the precipitation temperature of Al_3_Ni and Al_2_Cu achieved little influence (Figure 9c,d).

To further confirm the authenticity of the precipitation temperature of intermetallic compounds, a differential scanning calorimetry (DSC) analysis of four representative alloys was conducted. The DSC heating curves and the first derivative of the heat flow and temperature (noted as DH/dt) are as shown in Figure 9e–h. It is obvious that all the curves have a main sharp endothermic peak between 833 K and 873 K, which refers to the melting process of the matrix phases. It was concluded that the formation of most intermetallic compounds in experimental alloys concentrated on this temperature zone. And this is consistent with the results of the solidification paths in Figure 9a–d. With regard to the Al-12Si-1Cu-0.5Ni-xFe (x = 0.25 or 1.5) alloys in Figure 9e,f, the variations in the Fe content had little effect on the temperature that located the sharp endothermic peak. This phenomenon also applies to the alloys with the addition of Mn, as shown in Figure 9g,h. Differences in the DSC heating curves, caused by the variations in the phase composition in the different alloys and the corresponding temperature of the inflection point on the curves of DH/dt, are related to the formation temperature of intermetallics. It can be seen from a comparison of Figure 9e–h that more temperature-fluctuation regions appeared in the alloys with the addition of the Mn element, that is, more precipitated phases were formed compared with the alloys without Mn. This is consistent with the results in Table 5.

Further, the phase diagrams of the different alloys and the positions of key points were calculated and recorded, as demonstrated in Figure 10. By systematically analyzing the phase diagrams and verifying the related results obtained above, the phase-transition reactions of the alloys for the different stages of the solidification process were deduced as the results in Table 5. It can be seen that in the region with an Fe content of 0 to 2.0 wt.%, the phase transition is mainly a eutectic reaction and peritectic reaction, and the phases are all precipitated from a liquid phase or a primary phase at different stages during the solidification process with a decreasing temperature.

As indicated in Figure 10a, when the Fe content of the alloys without Mn elements was lower than Point 1 (0.3256 wt.%), *α*-Al and the Si phase (monatomic silicon phase) first precipitated in the alloy, followed by the Fe-rich phase. During the solidification process, due to the preferential precipitation of the Si phase, the concentration of the Fe in the remaining liquid phase was much more than that of the Si element, so that the Fe-rich phase was mainly *α*-Al_8_Fe_2_Si at that moment. And the precipitation temperature of *α*-Al_8_Fe_2_Si was lower than the Si phase. When the Fe content was at Point 1, *α*-Al_8_Fe_2_Si and the Si phase began to precipitate simultaneously at the same temperature. Moreover, in the Fe content region between Point 1 and Point 2 (0.3704 wt.%), the alloys first precipitated as *α*-Al, and then *α*-Al_8_Fe_2_Si began to appear on account of the decrease in Al and an increase in the proportion of the Fe element in the remaining liquid phase. Point 2 is the eutectic point in the phase diagram in Figure 10a, where *α*-Al, *α*-Al_8_Fe_2_Si, and *β*-Al_9_Fe_2_Si_2_ precipitated synchronously. In the alloys with an Fe content lower than Point 2, due to limitations in the solidification temperature range and microstructure growth space, it was difficult for the Fe-rich phase to continue to grow, and it eventually became skeleton- and fibrous-shaped, with a higher curvature distributed uniformly in the matrix, as shown in the Figure 3 and Figure 6. When the Fe content of the alloys were in the range of Point 2 to 2.0 wt.%, as shown in Figure 10a, the Fe proportion was high in the liquid phase, so that the Fe-rich phase first precipitated from the liquid phase with a decrease in the temperature, followed by the appearance of *α*-Al and the Si phase. At this time, the Fe-rich phases were dominated by *β*-Al_9_Fe_2_Si_2_, whose precipitation temperature could be much higher than *α*-Al. In this condition, the *β*-Al_9_Fe_2_Si_2_ phase had a sufficient solidification temperature interval and microstructure space to grow, and it eventually became a plate-shaped Fe-rich phase with a large length and without a curvature (Figure 3 and Figure 6). The above phase-transformation process explains, in detail, the evolutionary mechanism of Fe-rich phases in alloys without the addition of Mn that is caused by different Fe contents.

Similarly, in Figure 10b,c, the preferentially precipitated formation region of *α*-Al greatly compressed to within the new Point 2 (0.0869 wt.%) by adding the 0.35 wt.% Mn element. That is, only when the iron content was lower than Point 2 could *α*-Al be preferentially formed, and finally, *α*-Al_15_(FeMn)_3_Si_2_ appeared. At this moment, the atomic ratio of Fe and Mn in the *α*-Al_15_(FeMn)_3_Si_2_ phase was relatively low, at about 1:1 to 2:1. On the contrary, the Fe-rich phase would form first, if the Fe content increased to a higher value than the new Point 2 in Figure 10b,c. Specifically, when the content of Fe was in the region of Point 2 to Point 4 (0.6012 wt.%), the fractions of the iron elements in the liquid phase were relatively low, and *α*-Al_15_(FeMn)_3_Si_2_, containing a higher Mn content, was preferably formed first. As the temperature continued to decrease, the *β*-Al_15_(FeMn)_3_Si_2_ phase, containing lower Mn elements, the *α*-Al phase, and the Si phase precipitated. In this process, *β*-Al_15_(FeMn)_3_Si_2_ appeared later than the other phases, and it was difficult for it to grow due to limitations in the temperature range and growth space. As a result, the Fe-rich phase mainly presented as Chinese-character and petal-shaped. When the Fe contents of the alloys were between Point 4 and Point 5 (1.4792 wt.%), with an increase in the Fe content, the phase fraction ratio of *β*-Al_15_(FeMn)_3_Si_2_ to *α*-Al_15_(FeMn)_3_Si_2_ gradually increased. Additionally, at the position of Point 5, *β*-Al_15_(FeMn)_3_Si_2_ and *α*-Al_15_(FeMn)_3_Si_2_ precipitated at the same time and temperature. When the Fe content was at Point 5 to 2.0 wt.%, the *β*-Al_15_(FeMn)_3_Si_2_ with a lower Mn content preferentially formed due to the excessive Fe content and large fraction ratio of the Fe and Mn in the liquid phase. Then, the heavy consumption of the Fe element decreased the fraction ratio of Fe and Mn as the temperature dropped, leading to the formation of *α*-Al_15_(FeMn)_3_Si_2_. Similarly, the appearance of *α*-Al_15_(FeMn)_3_Si_2_ sharply reduced the Fe content fraction in the remaining liquid phase, resulting in an effective limitation in the continuous growth of *β*-Al_15_(FeMn)_3_Si_2_. Moreover, *α*-Al_15_(FeMn)_3_Si_2_ tended to accumulate in places where the *β*-Al_15_(FeMn)_3_Si_2_ phase grew too quickly. This was because the iron content in this region reduced more drastically than in other regions and was more suitable for *α*-Al_15_(FeMn)_3_Si_2_ precipitation. Moreover, the increasing formation of *α*-Al_15_(FeMn)_3_Si_2_ also effectually inhibited the growth of *β*-Al_15_(FeMn)_3_Si_2_ and made its growth direction deflect and caused it to have a certain curvature. In addition, the large consumption of Fe atoms by *α*-Al_15_(FeMn)_3_Si_2_ made it difficult for *β*-Al_15_(FeMn)_3_Si_2_ to aggregate and grow, which is equivalent to the phenomenon of fragmentation and refinement (Figure 4 and Figure 7). An analysis of these phase-transition reactions can systematically elaborate the modification mechanism of Mn on the microstructural evolution and solidification behaviors of Al-Si-based alloys with a high iron content.

### 4.2. Perspective of Solute Diffusion for Solidification Behaviosr in Different Alloys

**Figure 11:**
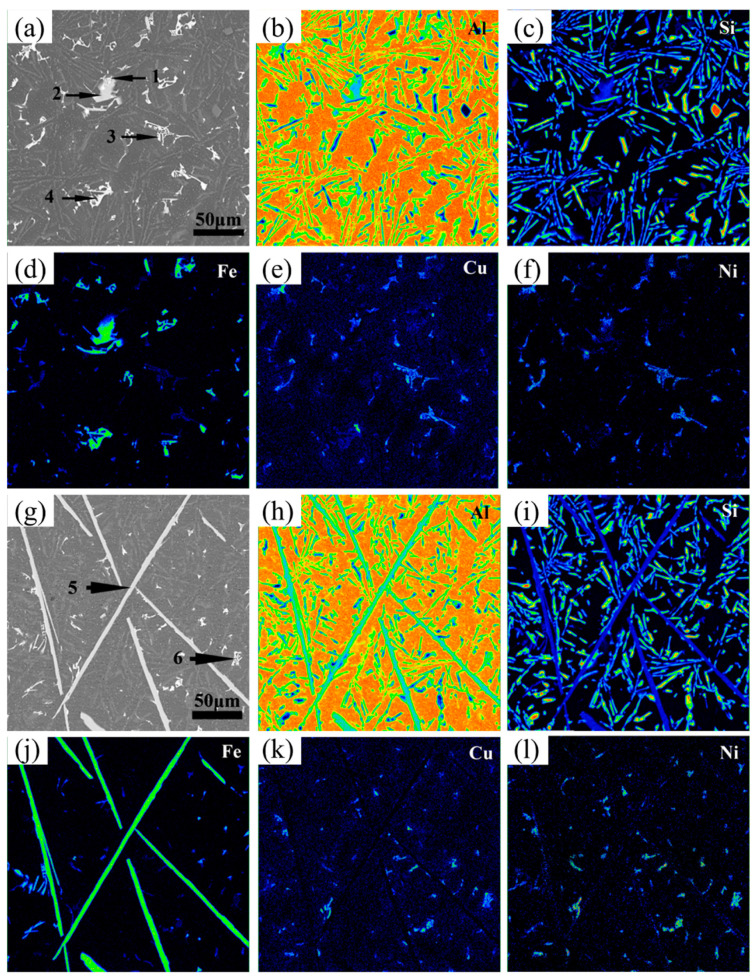
Map scanning images of Al-12Si-1Cu-0.5Ni-xFe alloys. (**a**–**f**) are the images of COMPO, Al, Si, Fe, Cu, and Ni in 0.25 wt.% Fe content alloys. (**g**–**l**) are the images of COMPO, Al, Si, Fe, Cu, and Ni in 2.0 wt.% Fe content alloys. Note: The (**1**–**6**) in (**a**) and (**g**) represent the Fe–rich phases with different shapes, respectively.

**Figure 12:**
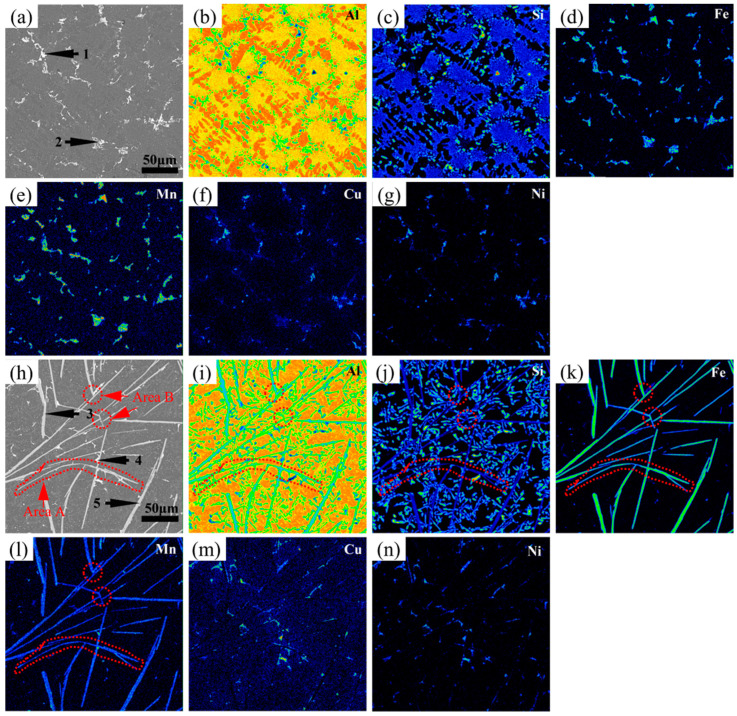
Map scanning images of Al-12Si-1Cu-0.5Ni-0.35Mn-xFe alloys. (**a**–**g**) are the images of COMPO, Al, Si, Fe, Mn, Cu, and Ni in 0.25 wt.% Fe content alloys. (**h**–**n**) are the images of COMPO, Al, Si, Fe, Mn, Cu, and Ni in 2.0 wt.% Fe content alloys. Note: The (**1**–**5**) in (**a**,**h**) represent the Fe–rich phases with different shapes, respectively. The the red dashed line frames in Area A and Area B represent the positions where the fracture is easy to occur during the growth of Fe–rich phases.

**Figure 13:**
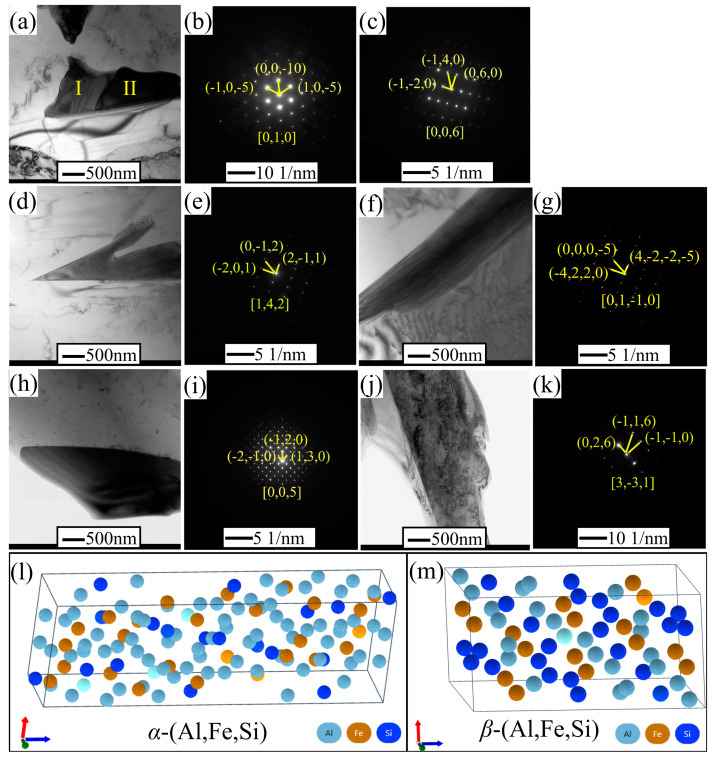
TEM results of different alloys and crystal structures of Fe-rich phases. (**a**,**d**,**f**) are TEM bright-field images in Al–12Si–1Cu–0.5Ni–0.25Fe alloys. (**h**,**j**) are TEM bright-field images in Al–12Si–1Cu–0.5Ni–0.35Mn–0.25Fe alloys. (**b**) and (**c**) are the SAED patterns marked I and II in (**a**), respectively. (**e**), (**g**), (**i**), and (**k**) are the SAED patterns that correspond to (**d**), (**f**), (**h**), and (**j**), respectively. (**l**) and (**m**) are the crystal structure of *α*–(Al,Fe,Si) and *β*–(Al,Fe,Si), respectively.

In order to further analyze the mechanism of the influence of solute diffusion and atomic aggregation capabilities on the solidification behaviors and the morphological evolution of Fe-rich phases, map scanning was carried out on the different alloys to obtain the distribution of each element in the microstructure, as shown in Figure 11 and Figure 12. Furthermore, TEM tests were performed to determine the specific phase composition of the alloys, as shown in Figure 13. It can be seen from Figure 11 that the microstructure was mainly composed of a matrix *α*-Al, a monatomic silicon phase, a binary Al-Si eutectic phase, an (Al,Cu,Ni) phase, and an Fe-rich phase. The content of the Si element in the Fe-rich phase was less than that in the Al-Si eutectic phase. It was also determined that the (Al,Cu,Ni) phase tended to attach to the Fe-rich phase. This is because the Cu and Ni elements enriched near the region of the Fe-rich phase form and grow quickly in the remaining liquid phase, due to the large consumption of the Al, Fe, and Si elements. In addition, the distribution of the Al and Si in Figure 11 indicates that the binding ability of the (Al,Si) phase is better than that of (Al,Fe,Si). Meanwhile, the bonding ability of Al-Cu and Al-Ni is similar, so they become the polymer precipitated phase of the (Al,Cu,Ni) phase [42]. Similar results can be seen in Figure 12. However, when the Mn was added, the binding ability of Al-Fe-Mn-Si became significantly stronger than that of Al-Fe-Si, so it is easier to form the (Al,Fe,Mn,Si) phase [39]. By comparing Figure 11 and Figure 12, it can also be seen that the Fe-rich phase becomes more refined after adding the manganese element. In alloys with a higher iron content, the addition of manganese can effectively produce a certain curvature of the Fe-rich phase (*β*-Al_15_(FeMn)_3_Si_2_), as shown in Area A of Figure 12h. In addition, it is obvious that the long Fe-rich phase with a large aspect ratio can be effectually reduced and broken into shorter phases by adding the Mn element. And the formation of *α*-Al_15_(FeMn)_3_Si_2_ containing more manganese clearly appears at the broken site, as shown in Area B of Figure 12h.

For further verifying the existence of the phases in alloys, high-resolution transmission electron microscopy was performed for the Al-12Si-1Cu-0.5Ni-0.25Fe and Al-12Si-1Cu-0.5Ni-0.25Fe-0.35Mn alloys. And the results, including the representative bright-field (BF) TEM morphologies and the selected area electron diffraction (SAED) patterns demonstrated in Figure 13, were used to identify the crystal structures of the constituent phases. The images confirm that the SAED marked I in Figure 13a is the (Al,Cu,Ni) phase (Figure 13b), while the region marked II is the Al_3_Fe presented as a triclinic system (Figure 13c), and these still remain in the target alloys. Additionally, it can be deduced that *β*-Al_9_Fe_2_Si_2_ presents as a monoclinic system (Figure 13d,e,m) and *α*-Al_8_Fe_2_Si presents as a hexagonal system (Figure 13f,g,l). The *α*-Al_15_(FeMn)_3_Si_2_ presents as a cubic system with a high Mn atomic fraction (Figure 13h,i), while *β*-Al_15_(FeMn)_3_Si_2_ presents as a monoclinic system with a low Mn atomic fraction (Figure 13j,k), similar to *β*-Al_9_Fe_2_Si_2_. Therefore, it is evident that the existing phases in the experiment alloys are mainly the *α*-Al, Si eutectic, (Al,Cu,Ni), *α*-Al_8_Fe_2_Si, and *β*-Al_9_Fe_2_Si_2_ phases in the alloys without the addition of Mn. And the addition of the Mn element can transform the (Al,Fe,Si) phases to Al_15_(FeMn)_3_Si_2_, whose atomic ratio of Fe and Mn is close to 1:1 and 5:1, respectively.

According to previous research [37,43,44], it should be noted that the binary Al-Fe phases in the solidified structure are derived from the initial Al-Fe master alloy and can cause the evolution of the *α*-Al_8_Fe_2_Si and *β*-Al4.5FeSi phases during solidification [18]. However, Al-Fe can only be observed from the XRD patterns (Figure 5) and the TEM results (Figure 13c) because of the low content. In addition, some studies have shown that the level of the Si content in Al alloys seems to be critical in determining the formation of the Al-Al_3_Fe eutectic phase during solidification [44]. Combining the calculated equilibrium phase diagrams (Figure 10a and Table 5) with the XRD analysis results (Figure 5), it can easily be seen that that when the Fe content is lower than 0.3704 wt.% (Point 2 in Figure 10a), *α*-Al_8_Fe_2_Si will form by sequential reactions from the remaining liquid phase. It is well known that a series of atom diffusions can occur inevitably in an alloy melt, as new phases are formed following solidification. Owing to the partition coefficient of Fe in the Al-based liquid phase being quite small at 0.022, there will be a high degree rejection of Fe atoms at the solidification frontier. That is to say, solute diffusion exists at the interface between the growing primary *α*-Al phase and the liquid during solidification, and Fe atoms will be enriched at the solidification frontier [44,45]. As a result, the abnormal clustering tendencies of Al and Fe atoms in the liquid ahead of the solidifying interface will trigger nucleation. When the primary *α*-Al (face-centered cubic crystal structure) phase first precipitates with the form of dendrites, the diffusion of the Fe and Si atoms begins to emerge at the solid–liquid interface. Based on the minimum energy principle and taking the factors of thermodynamics and kinetics into account, the liquid phase can react with the Al_3_Fe phase and can transform into the *α*-Al_8_Fe_2_Si phase in the Al-12Si-1Cu-0.5Ni-xFe system following the solidification path instantly and spontaneously [43,44]. Subsequently, the large amount of the *α*-Al_8_Fe_2_Si-phase formation will lead to the enrichment of the Si atom at the interface front of the solid–liquid, so that the precipitation of *β*-Al_9_Fe_2_Si_2_ is promoted to consume the excess silicon. That is, the evolution from the Al2Fe phase to *α*-Al_8_Fe_2_Si occurs instantly in the liquid, followed by the formation of *β*-Al_9_Fe_2_Si_2_ due to the diffusion of the Fe and Si atoms at the solid–liquid interface. However, when the Fe content is much higher than 0.3704 wt.% (Point 2 in Figure 10a), *β*-Al_9_Fe_2_Si_2_ will preferentially form instead of *α*-Al_8_Fe_2_Si. With the formation and growth of *β*-Al_9_Fe_2_Si_2_, the Al and Si atoms are largely consumed, while some Fe atoms are enriched and diffused, thus promoting the appearance of *α*-Al_8_Fe_2_Si, which consumes the excess Fe atoms. A similar reaction sequence also occurs in the Al-12Si-1Cu-0.5Ni-0.35Mn-xFe system during solidification. When the Fe content is between 0.0869 wt.% (Point 2 in Figure 10b) and 1.4792 wt.% (Point 5 in Figure 10b), the evolution from the Al_2_Fe phase to *α*-Al_15_(FeMn)_3_Si_2_, containing more Mn elements and having a lower atomic ratio of Fe and Mn of close to 1 to 1 or 2 to 1, can occur instantly and preferentially in the liquid phase, due to the stronger atomic aggregation capabilities of Al, Fe, Mn, and Si. Subsequently, the excess Si and Fe atoms and the trace amounts of Mn atoms will experience reactions with the residual liquid phase and will form *β*-Al_15_(FeMn)_3_Si_2_, containing less Mn elements and having a higher atomic ratio of Fe and Mn of close to 5 to 1. Additionally, due to the precipitation of *α*-Al and *α*-Al_15_(FeMn)_3_Si_2_ and the consumption of considerable Al atoms, the Si atoms are enriched at the front of the solid–liquid interface and precipitate the silicon phase in the residual melt. This is followed by the eutectic reaction of Al-Si, as well as the formation of an Al-Si eutectic system with a short acicular morphology. Herein, it is worth noting that the primary phase will continue to grow during the following solidification process, and it will be accompanied by the precipitation of subsequent phases and will tend to form dendrites. As a result, most of the Cu and Ni atoms can be blocked between Fe-containing intermetallic and Si eutectic systems, owing to limitations in time and space for them to diffuse uniformly due to their low solubility and diffusion coefficients [9,10]. Therefore, it is very common for (Al,Cu,Ni) precipitates to appear close to Fe-rich phases. The aforementioned results are in accordance with the results of the equilibrium phase diagram regarding the evolution of Fe-rich phases. It should also be noted that the addition of Mn has the ability to change Fe-rich intermetallics from *β*-Fe-rich phases with a platelet-like shape to *α*-Fe-rich phases [46,47,48]. However, the neutralizing ability of the addition of Mn is limited and cannot impede the preferential formation of *β*-Al_15_(FeMn)_3_Si_2_ containing a smaller proportion of Mn atoms when the large amount of Fe content is between 1.4792 wt.% and 2.0 wt.% in the alloys [49]. With regard to the relationship between Fe and Mn, some of the literature has considered that the manganese content should not be less than half of the iron, when the Fe content exceeds 0.45% [50]. The results achieved by Glaisher on a series of Al-5% Si alloys show that segregation can be caused by the ratio of Fe and Mn being 1.3 and 3.8, respectively [31]. It can be clear that the morphology of intermetallic compounds is consistent with the experimental results, with respect to the formation of a refined *α*-Fe-rich phase and a platelet-like *β*-Fe-rich phase, as shown in Figure 3 and Figure 4. Ashtari suggests that when the ratio of Fe and Mn is 3.57, partial substitution from the *β*-Fe-rich phase to a less-detrimental *α*-Fe-rich phase can be promoted, although a significant amount of the *β* phase will be formed. This discovery corresponds to the research results shown in Figure 3, Figure 4 and Figure 5.

Noteworthily, in this study, through systematic analyses and research, it can be concluded that manganese atoms can replace iron atoms in Fe-rich phases, thus causing an evolution and resolution of them. The main reason for this is that manganese atoms have an atomic radius and crystal structure (a body-centered cubic structure) that are very close to those of iron atoms and have a stronger bonding ability with aluminum atoms. When the Fe content is lower than 1.5 wt.%, the proportion of Mn is relatively high. In this process, the *α*-Fe-rich phase can precipitate earlier and more easily than the *β*-Fe-rich phase, leading to an effective limitation in the formation and growth of the *β*-Fe-rich phase by reducing the remaining time and space. Furthermore, when the Fe content is higher than 1.5 wt.%, the proportion of manganese is comparatively small. In this case, the fact that a large number of iron elements preferentially form the *β*-Fe-rich phase containing a small amount of manganese atoms cannot be avoided and prevented. As the *β*-Fe-rich phase continually forms and grows, plenty of iron atoms are consumed. At this point, there will be a large amount of manganese atoms in the solidification front and the growth front of the *β*-Fe-rich phase, resulting in a sharp decrease in the nucleation and growth ability of the *β*-Fe-rich phase. Additionally, the growth direction of the *β*-Fe-rich phase will also be deflected due to solute diffusion and enrichment and will form a shape with a certain curvature. At this moment, the *α*-Fe-rich phase will begin to appear in the fracture of the *β*-Fe-rich phase.

In general, without the addition of Mn, an increase in the iron content will affect the chemical reaction and precipitation sequence of the alloys, resulting in a morphological change of the Fe-rich phase, becoming plate-like in shape with large length and aspect ratio and with no curvature, which seriously damages the properties of the alloy. Importantly, adding a 0.35 wt.% Mn element to Al-12Si-1Cu-0.5Ni-xFe alloys can effectively increase the iron content threshold required for the formation of the *β*-Fe-rich phase from 0.3704 wt.% without the addition of Mn to about 1.5 wt.%. When the iron content does not exceed 1.5 wt.%, a *α*-Fe-rich phase will be preferentially formed, limiting the formation and growth of the *β*-Fe-rich phase from the perspective of space and time. When the iron content reaches the region of 1.5 wt.% to 2 wt.%, the addition of manganese can still limit the continuous growth of the *β*-Fe-rich phase from the perspective of solute diffusion and can achieve the effect of refining the Fe-rich phase. This manuscript mainly focuses on the research on the mechanism of the phase-transition reactions, solute diffusions, solidification behaviors, and phase-formation and -growth process of alloys with different additions of Mn in different Fe contents, especially a high content of 2.0 wt.%. Theoretically speaking, the properties of alloys with optimized iron-rich phases were effectively improved. In this paper, due to the focus on the study of mechanisms, the improvement law of performance will be further highlighted in subsequent research.

## 5. Conclusions

In this paper, the microstructural evolution and solidification behaviors of Al-12Si-1Cu-0.5Ni with different contents of Fe and Mn elements were studied comprehensively, and the mechanisms were systematically deduced by combining experiments with simulations. Accordingly, the following conclusions can be drawn:

(1) The addition of Fe varies the phase-transition reactions and precipitation sequence and forms Fe-rich phases. In alloys with a lower Fe content than 0.25 wt.%, *α*-Al first precipitates, followed by *α*-Al_8_Fe_2_Si. Increasing the Fe content to above 0.37 wt.%, *β*-Al_9_Fe_2_Si_2_ preferentially forms with a larger phase fraction than *α*-Al_8_Fe_2_Si, as well as a higher precipitation temperature than *α*-Al. The addition of 0.35 wt.% Mn effectively transforms the Fe-rich phases to Al_15_(FeMn)_3_Si_2_, which is mainly *α*-Al_15_(FeMn)_3_Si_2_ with an atomic ratio of Fe and Mn 1:1 when the Fe content ranges from 0.09 wt.% to 0.60 wt.%. The threshold of the Fe content required for the preferential precipitation of *β*-Al_15_(FeMn)_3_Si_2_ with an atomic ratio of Fe and Mn of 5:1 can be increased to 1.48 wt.%.
(2) In alloys without Mn, by increasing the Fe content, the morphology of the Fe-rich phase changes from a skeletal shape (0.1–0.25 wt.%) to a fibrous shape with curvatures (0.5 wt.%) and then to a needle-like (1.0 wt.%) and plate-like shape with no curvature (2.0 wt.%). The maximum length and mean aspect ratio increase from 12.01 μm to 655.66 μm and from 1.96 to 84.05, and the mean curvature decreases from 8.66 × 10^−2^ μm^−1^ to 8.25 × 10^−4^ μm^−1^. The addition of Mn makes the Fe-rich phases surface as a Chinese-character and petal shape when the Fe content is lower than 0.5 wt.%, while they transform to a broken and refined plate-like shape with a certain curvature in alloys whose Fe content increases to 2.0 wt.%. Ultimately, under the same Fe-content conditions, the maximum length and the aspect ratio can be effectively reduced to 46.2% and 42.0%, respectively, while the curvature can be noticeably increased by 3.27 times with the addition of Mn.
(3) Mn can modify Fe-rich phases by changing the phase reactions and increasing the threshold of the Fe content required for the precipitation of the *β*-Fe-rich phases. Therefore, the formation and growth of *β*-Al_15_(FeMn)_3_Si_2_ can be effectively restricted simultaneously in time and space. Moreover, the enrichment of Mn atoms and the solute diffusion at the solidification front and the growth front of *β*-Al_15_(FeMn)_3_Si_2_, as well as the strong atomic binding ability, cause the growth direction of *β*-Al_15_(FeMn)_3_Si_2_ to deflect with a certain curvature. Additionally, the enriched Mn atoms quickly form a *α*-Al_15_(FeMn)_3_Si_2_ phase and cause the long *β*-Al_15_(FeMn)_3_Si_2_ phase to be broken and refined to reduce the damages to the performance and circularity of alloys.

## Figures and Tables

**Table 1:** Chemical compositions of samples calculated by XPS (wt.%).

No.	Al	Si	Cu	Ni	Fe	Mn
1#	86.39	12.00	1.00	0.50	0.10	-
2#	86.28	11.99	0.99	0.51	0.25	-
3#	86.03	12.01	0.99	0.49	0.50	-
4#	85.49	12.01	1.00	0.50	1.00	-
5#	85.14	12.00	1.00	0.50	1.50	-
6#	84.43	11.99	0.99	0.50	2.00	-
7#	86.19	11.99	1.00	0.49	0.10	0.35
8#	85.82	12.00	1.00	0.51	0.25	0.35
9#	85.42	12.00	1.01	0.50	0.50	0.35
10#	85.19	12.01	1.01	0.50	1.00	0.35
11#	84.73	12.01	1.00	0.50	1.50	0.35
12#	84.09	12.00	0.99	0.50	2.00	0.35

**Table 2:** Solidification sequence in different samples correspond to Figure 1 and Figure 2.

No.	Mn Content (wt.%)	Fe Content (wt.%)	Precipitation Temperature, K, and Phase Fraction (%)				
			α-Al	(Al,Si)	(Al,Ni)	(Al,Si,Fe)	(Al,Si,Fe,Mn)
1#	0	0.10	850.31, 88.21	846.90, 10.78	814.62, 0.68	842.17, 0.32	-
2#		0.25	849.87, 87.80	846.58, 10.71	814.60, 0.68	845.70, 0.80	-
3#		0.50	849.61, 87.12	846.37, 10.58	814.58, 0.68	862.28, 1.61	-
4#		1.00	850.10, 85.75	846.30, 10.32	814.55, 0.69	893.73, 3.23	-
5#		1.50	850.61, 84.37	846.22, 10.07	814.50, 0.70	913.50, 4.86	-
6#		2.00	851.12, 82.98	846.13, 9.81	814.46, 0.71	928.22, 6.51	-
7#	0.35	0.10	850.15, 87.41	846.31, 10.72	814.52, 0.69	-	852.19 1.18
8#		0.25	849.86, 86.97	846.29, 10.68	814.54, 0.69	-	867.76 1.66
9#		0.50	849.38, 86.29	846.03, 10.58	814.46, 0.69	845.22 0.41	882.76 2.03
10#		1.00	849.58, 84.91	846.31, 10.33	814.52, 0.70	888.16 2.03	901.26 2.03
11#		1.50	850.08, 83.52	845.96, 10.07	814.42, 0.70	914.29 3.66	913.66 2.04
12#		2.00	850.59, 82.13	845.87, 9.81	814.38, 0.71	928.96 5.31	915.33 2.04
13#	0	0	850.61 88.48	847.20 10.83	814.76 0.68	-	-

Note: #13 is the comparative group without the addition of the Fe and Mn elements.

**Table 3:** EDS results of Al-12Si-1Cu-0.5Ni-xFe (x = 0~2.0 wt.%) alloys in Figure 3 (at.%).

Point	Al	Si	Cu	Fe	Ni
1	70.11	18.13	0.92	8.52	2.32
2	61.37	0.31	18.56	0.60	19.16
3	45.66	53.57	0.26	0.24	0.27
4	68.12	21.80	0.93	9.03	0.12
5	1.21	98.15	0.15	0.20	0.29
6	64.42	0.35	16.53	1.38	17.32
7	68.08	22.10	0.12	9.35	1.35
8	68.11	17.31	0.76	11.59	2.23
9	59.99	0.32	19.23	0.79	19.67
10	67.20	18.56	0.68	12.50	1.05
11	67.13	18.52	0.76	12.39	1.19
12	67.07	18.66	0.93	12.22	1.12
13	67.15	18.73	0.81	12.24	1.07
14	62.42	0.88	17.85	1.81	17.04

**Table 4:** EDS results of Al-12Si-1Cu-0.5Ni-0.35Mn-xFe (x = 0~2.0 wt.%) alloys in Figure 4 (at.%).

Point	Al	Si	Cu	Fe	Ni	Mn
1	71.28	11.86	1.34	7.55	1.21	6.76
2	62.42	0.29	17.85	1.07	18.04	0.33
3	73.91	9.40	2.19	7.34	1.83	5.33
4	69.91	10.93	1.10	10.60	1.01	6.45
5	70.95	10.54	0.87	10.24	0.91	6.49
6	65.69	20.19	0.18	11.06	0.29	2.59
7	71.27	11.14	0.92	9.05	0.60	7.02
8	65.91	18.50	0.43	11.80	0.94	2.42
9	65.69	20.19	0.18	11.06	0.29	2.59

**Table 5:** Phase-transition reactions with decreasing temperatures of Al-12Si-1Cu-0.5Ni-xFe and Al-12Si-1Cu-0.5Ni-0.35Mn-xFe alloys (x = 0~2.0 wt.%) correspond to the phase diagrams in Figure 10.

Mn Content	Fe Content	Phase-Transition Reactions with Decreasing Temperatures
0 wt.%	0 wt.%—P1	L → L + *α*-Al → L + *α*-Al+ Si → L + *α*-Al + Si + *α*-AFS
	P1	L → L + *α*-Al → L + *α*-Al + Si + *α*-AFS
	P1–P2	L → L + *α*-Al → L + *α*-Al + *α*-AFS → L + *α*-Al + Si + *α*-AFS
	P2	L → L + *α*-Al + *α*-AFS + *β*-AFS → L + *α*-Al + *α*-AFS + *β*-AFS + Si
	P2—2 wt.%	L → L + *β*-AFS → L + *β*-AFS + *α*-AFS + *α*-Al → L + *β*-AFS + *α*-AFS + *α*-Al + Si
0.35 wt.%	0 wt.%—P1	L → L + *α*-Al → L + *α*-Al + Si → L + *α*-Al + Si + *α*-AFMS
	P1	L → L + *α*-Al → L + *α*-Al + Si + *α*-AFMS
	P1–P2	L → L + *α*-Al → L + *α*-Al + *α*-AFMS → L + *α*-Al + *α*-AFMS + Si
	P2	L → L + *α*-Al + *α*-AFMS → L + *α*-Al + *α*-AFMS + Si
	P2–P3	L → L + *α*-AFMS → L + *α*-AFMS + *α*-Al → L + *α*-AFMS + *α*-Al + Si
	P3	L → L + *α*-AFMS → L + *α*-AFMS + *α*-Al → L + *α*-AFMS + *α*-Al + *β*-AFMS + Si
	P3–P4	L → L + *α*-AFMS → L + *α*-AFMS + *α*-Al → L + *α*-AFMS + *α*-Al + *β*-AFMS → L + *α*-AFMS + *α*-Al + *β*-AFMS + Si
	P4	L → L + *α*-AFMS→L + *α*-AFMS + *α*-Al + *β*-AFMS → L + *α*-AFMS + *α*-Al + *β*-AFMS + Si
	P4–P5	L → L + *α*-AFMS → L + *α*-AFMS + *β*-AFS → L + *α*-AFMS + *β*-AFMS + *α*-Al → L + *α*-AFMS + *β*-AFMS + *α*-Al + Si
	P5	L → L + *α*-AFMS + *β*-AFMS → L + *α*-AFMS + *β*-AFMS + *α*-Al → L + *α*-AFMS + *β*-AFMS + *α*-Al + Si
	P5—2 wt.%	L → L + *β*-AFMS → L + *β*-AFMS + *α*-AFMS → L + *β*-AFMS + *α*-AFMS + *α*-Al → L + *β*-AFMS + *α*-AFMS + *α*-Al + Si

Note: L denotes Liquid; AFS denotes (Al,Fe,Si) phases; AFMS denotes (Al,Fe,Mn,Si) phases; and P denotes Point in the phase diagrams in Figure 10, respectively.

## Data Availability

Data is contained within the article, and no new data were created or analyzed in this study.
